# Factors associated with changes in quality of life after pancreaticoduodenectomy for periampullary tumors

**DOI:** 10.3389/fsurg.2026.1797159

**Published:** 2026-04-23

**Authors:** Dat Tien Le, Helal Metwalli, Long Cong Duy Tran, Nguyen Van Hai, Federica Cucè, Gam Hong Pham, Manar A. Balouz, Mohammed Eldoadoa, Hiba Hamdar, Hoang Viet Nguyen, Nguyen Tien Huy

**Affiliations:** 1 Department of Hepatobiliary and Pancreatic Surgery, University Medical Center at Ho Chi Minh City, Ho Chi Minh, Vietnam; 2 Department of Surgery, Faculty of Medicine, University of Medicine and Pharmacy at Ho Chi Minh City, Hồ Chí Minh, Vietnam; 3 Faculty of Medicine, Benha University, Banha, Egypt; 4 Online Research Club, Nagasaki, Japan; 5 Department of Engineering for Innovation Medicine, University of Verona, Verona, Italy; 6 Faculty of Pharmacy, Pham Ngoc Thach University of Medicine, Hồ Chí Minh, Vietnam; 7 Faculty of Medicine Menoufia University, Shibin el Kom, Egypt; 8 St Helens & Knowsley NHS Teaching Hospital Trust, Saint Helens, United Kingdom; 9 TsentarPlovdiv Center, Plovdiv Medical University, Plovdiv, Bulgaria; 10 Medical Learning Skills Academy, Beirut, Lebanon; 11 Can Tho University of Medicine and Pharmacy, Cần Thơ, Vietnam; 12 Institute of Research and Development, Duy Tan University, Da Nang, Vietnam; 13 School of Medicine and Pharmacy, Duy Tan University, Da Nang, Vietnam; 14 Graduate School of Tropical Medicine and Global Health (TMGH), Nagasaki University, Nagasaki, Japan

**Keywords:** pancreatectomy, pancreatic cancer, periampullary tumors, quality of life, SF-36

## Abstract

**Introduction:**

Pancreaticoduodenectomy for periampullary tumors is a complex surgery with potential complications and long recovery, impacting patients' quality of life (QoL). This study evaluates QoL changes from preoperative status to 12 months post-surgery.

**Methods:**

A prospective cohort study was conducted at the University Medical Center of Ho Chi Minh City from January 2019 to May 2021. Patients undergoing pancreaticoduodenectomy (laparoscopic or open) were assessed using the SF-36 questionnaire at baseline, 1, 3, 6 and 12 months post-discharge. Exclusion criteria included total pancreatectomy, in-hospital mortality and disease recurrence. Data on demographics, comorbidities, tumor staging, complications and survival were analyzed using SPSS 20.0, with paired Wilcoxon tests and regression models.

**Results:**

Among 111 patients (64 males, mean age 57 ± 12.2 years), 44 had pancreatic head cancer and 21 had ampullary cancer. Laparoscopic surgery was performed in 66 cases. Postoperative fistulas occurred in 30.6%, with one severe case. Median hospital stay was 11 ± 5 days, and average disease-free survival was 20.42 ± 13.4 months. QoL declined at 1 month post-surgery (Physical: 67.29 to 54.69, *p* = 0.04; and Emotional limitations: 70.17 to 54.18, *p* = 0.01) but improved from 3 months onward, surpassing pre-treatment levels. Malignancy, comorbidities, cholangitis and postoperative complications negatively impacted QoL.

**Conclusion:**

QoL decreases initially after pancreaticoduodenectomy but stabilizes and improves after 3 months. Preoperative and postoperative management of risk factors may enhance recovery and QoL outcomes.

## Introduction

Periampullary tumors represent a heterogeneous group of neoplasms arising from the pancreatic head, distal common bile duct, and duodenum, the majority of which are malignant. Their often insidious onset and nonspecific early manifestations lead most patients to present with advanced disease, when biliary or pancreatic duct obstruction, jaundice, pain, and nutritional compromise significantly impair health-related quality of life (HRQoL) and limit survival (1). Upon diagnosis, approximately 10% of patients present with early-stage disease amenable to potentially curative resection, 32% have locally advanced but non-metastatic tumors, and the remaining 57% have metastatic disease not suitable for surgical treatment (2). When resection is feasible, pancreaticoduodenectomy remains the cornerstone of curative management, often in combination with adjuvant chemotherapy and radiotherapy, while best supportive care is reserved for those with nonresectable disease. Prognosis after resection varies substantially according to histological subtype, tumor size, stage, resection margins, and patient-related factors such as functional reserve, comorbidities, and response to systemic therapy (3–5).

Although pancreaticoduodenectomy offers the best chance for long-term survival, it remains a complex surgical procedure associated with considerable perioperative morbidity, extended recovery, and potential long-term consequences for nutritional status and physical functioning. As perioperative outcomes have improved, attention has increasingly shifted toward patient-centered endpoints such as HRQoL, functional independence, and symptom burden. In oncologic and complex surgical practice, QoL is now recognized as a critical outcome alongside traditional measures of survival, reflecting patients' growing preference to evaluate treatment success not only in terms of longevity but also overall well-being (6, 7).

Despite its clinical importance, the literature on QoL after pancreaticoduodenectomy remains fragmented. Most studies focus on short-term recovery, with heterogeneous designs, limited follow-up, and variability in measurement instruments. Data assessing long-term trajectories across distinct QoL domains using standardized, validated tools is scarce. Furthermore, the influence of modifiable factors, such as comorbidities, cholangitis, surgical approach, and postoperative complications, on HRQoL recovery is not yet well characterized. Addressing these gaps is essential to guide perioperative counseling, optimize recovery pathways, and support individualized care strategies for patients undergoing pancreaticoduodenectomy for periampullary tumors (6–11).

This prospective cohort study therefore aims to evaluate changes in SF-36 HRQoL domains from the preoperative period to 12 months after pancreaticoduodenectomy for periampullary tumors and to identify clinical and perioperative factors associated with HRQoL over time, including comorbidities, cholangitis, tumor pathology, and postoperative complications.

## Methods

### Study design and setting

A prospective cohort study was carried out to health-related quality of life (HRQoL) in patients undergoing pancreaticoduodenectomy for periampullary tumors at the University Medical Center of Ho Chi Minh City. The population comprised adults with periampullary tumors considered for curative pancreaticoduodenectomy at the institution. All consecutive patients deemed suitable for pylorus-preserving pancreaticoduodenectomy after discussion at a multidisciplinary tumor board, between January 2019 and May 2021, were proposed to participate in the study. No formal prior sample size calculation was performed; the sample was determined by the number of eligible patients treated during the study period, and the study is therefore exploratory and hypothesis-generating with respect to effect estimates. Reporting followed the STROBE recommendations for observational studies, Supplementary Table S1 (12).

### Participants

Patients were eligible if they were 18 years of age or older, had a radiological diagnosis of a periampullary tumor arising from the pancreatic head, distal common bile duct, or duodenum, and were scheduled for elective pylorus-preserving pancreaticoduodenectomy with curative intent. Additional inclusion criteria were the ability to participate in an interviewer-administered questionnaire in the local language and provision of written informed consent. Exclusion criteria included current treatment with antidepressants or other psychotropic medications for previously diagnosed psychiatric disorders, the need for total pancreatectomy or additional organ resection beyond a standard pancreaticoduodenectomy, and intraoperative identification of unresectable disease leading to palliative bypass or biopsy instead of resection. Patients who died during the index hospital admission or who developed documented tumor recurrence during follow-up were excluded from the QoL analyses because recurrence was expected to be a major, independent determinant of HRQoL. All operations were performed by a single experienced hepatopancreatobiliary surgeon using a standardized pylorus-preserving pancreaticoduodenectomy technique, thereby reducing variability in surgical approach.

### Preoperative assessment and perioperative management

Preoperative evaluation followed a uniform institutional, all patients underwent pancreatic protocol computed tomography of the abdomen to characterize the primary lesion and vascular anatomy and chest computed tomography to exclude distant metastases. Tumor markers and routine laboratory tests were obtained, and comorbidities and nutritional status were systematically assessed. Preoperative biliary drainage was recommended for patients presenting with cholangitis or with total serum bilirubin levels exceeding 10 mg/dL, using either endoscopic retrograde biliary drainage or percutaneous transhepatic biliary drainage according to expertise and anatomy, in case these weren't feasible other drainage methods were adopted (14).

All cases were discussed in a multidisciplinary tumor board, and the decision for pancreaticoduodenectomy was made after considering tumor resectability, comorbid conditions, and patient preferences (15). In most cases, preoperative histological confirmation was not possible, and the indication for surgery was based on radiologic features suggestive of malignancy and multidisciplinary consensus, in line with international practice for resectable periampullary tumors. Patients received detailed counseling about the nature of the disease, the risks and benefits of surgery, the possibility of unresectable disease being found intraoperatively, and alternative treatment options, and provided written informed consent prior to surgery and study enrollment. Postoperative care, including analgesia, nutrition, and management of complications, followed institutional protocols based on established pancreatic surgery standards (16, 17).

### Quality of life assessment

HRQoL was assessed with the Short Form-36 Health Survey (SF-36), a widely validated generic questionnaire for measuring QoL in surgical and oncologic populations. The SF-36 comprises items grouped into eight domains: Physical Functioning (PF), Role Limitations due to Physical Health (RP), Bodily Pain (BP), General Health Perceptions (GH), Vitality (VT), Social Functioning (SF), Role Limitations due to Emotional Problems (RE), and Mental Health (MH), which can be combined into the Physical Component Summary (PCS) and Mental Component Summary (MCS) scores. Each domain score was transformed to a 0–100 scale, with higher scores reflecting better health status and functioning. Interpretation of SF-36 scores is reported in Supplementary Table S2. A Vietnamese version of the SF-36, previously validated, was administered by trained research staff through face-to-face interviews to minimize missing data and ensure consistent interpretation of items (18). Standard SF-36 scoring algorithms were applied; if responses to more than half of the items in a given domain were missing, the corresponding domain score was treated as missing, whereas domains with fewer missing items were scored according to the manual rules (19–22).

Scores were derived using the recommended weighted aggregation algorithm based on standardized domain scores. While individual domain scores were transformed to a 0–100 scale, summary scores are not strictly bounded within this range, which should be considered when interpreting values exceeding 100.

Quality of life was evaluated at five predefined time points. Baseline assessment (V0) was performed after diagnosis and treatment planning and, when preoperative biliary drainage was necessary, after biliary decompression and prior to surgery. Postoperative assessments were conducted at 1 month (V1), 3 months (V3), 6 months (V6), and 12 months (V12) after hospital discharge, coinciding with routine surgical follow-up visits. All interviews were carried out in person in the outpatient clinic by investigators who were trained in questionnaire administration and who followed a standardized script. When patients were hospitalized for postoperative complications, follow-up interviews were performed at the scheduled time points and were not altered by the rehospitalization itself; in such cases, the total length of hospital stay was defined as the sum of the index admission and readmission durations. To account for loss to follow-up increase over time, particularly after 6 and 12 months; analyses of HRQoL changes at each time point were therefore conducted on a complete-case basis, including only patients with available SF-36 data at the corresponding time, and no imputation of missing QoL data was performed (21, 22).

### Clinical data collection

Clinical data were collected prospectively from structured interviews and electronic medical records in the hepatobiliary and pancreatic surgery department. Baseline variables included demographic information (age, sex, residence, and occupation), admission indication (such as jaundice, abdominal pain, or other symptoms), comorbidities, body mass index, and the need for preoperative nutritional support. Tumor-related variables comprised imaging findings, final histopathological diagnosis, local tumor extent including vascular or surrounding tissue invasion, and, when available, pathological staging. Perioperative data encompassed surgical approach (laparoscopic vs. open), operative details, postoperative complications, length of stay, and readmissions within the early postoperative period. Postoperative pancreatic fistula and post-pancreatectomy hemorrhage were defined and graded according to the International Study Group of Pancreatic Surgery criteria (23, 24), and overall complications were classified using the Clavien–Dindo grading system (25, 26). Oncologic outcomes included receipt of adjuvant chemotherapy, tumor recurrence during follow-up, and overall and disease-free survival, with survival time calculated from the date of diagnosis to the date of last follow-up or death. All data were entered into a secure, anonymized electronic database with restricted access to the study team.

### Statistical analysis

Statistical analyses were conducted using SPSS software, version 20.0 (IBM Corp, Armonk, NY, USA). Categorical variables were summarized as counts and percentages, and continuous variables as means with standard deviations or medians with ranges or interquartile ranges, depending on their distribution. The distribution of continuous variables was evaluated using skewness and kurtosis statistics. For comparisons between independent groups, Student's t test was used for normally distributed continuous variables and the Mann–Whitney *U*-test for non-normally distributed variables, while chi-square or Fisher exact tests were applied for categorical variables according to cell counts (27, 28).

To evaluate changes in HRQoL over time, SF-36 domain and summary scores at V0, V1, V3, V6, and V12 were compared using the paired Wilcoxon signed-rank test, and results are reported as medians with interquartile ranges. When comparing means across more than two groups, analysis of variance or its robust alternative was used depending on the result of Levene's test for homogeneity of variances, and Bonferroni or Tamhane's T2 procedures were applied for *post hoc* pairwise comparisons where appropriate. Univariable and multivariable generalized regression models were built to explore associations between clinical variables and HRQoL scores (29). In these models, SF-36 scores or score sums were treated as continuous dependent variables, and demographic, clinical, and tumor-related factors, such as age group, sex, residence, occupation, admission indication, comorbidities, body mass index, nutritional support, preoperative cholangitis, and final pathology, were included as potential predictors. In addition, binary logistic regression analyses were performed for each SF-36 domain, with impaired HRQoL status in that domain as the dependent variable and the same set of clinical covariates as explanatory variables. Variables with a *p* value less than 0.20 in univariable analyses were considered for inclusion in multivariable models, which were fitted using a backward stepwise selection procedure with a significance level of 0.05 for retention. The number of predictors included in the final models was constrained by the sample size and number of outcome events to reduce the risk of overfitting, and multicollinearity was assessed through inspection of variance inflation factors. All hypothesis tests were two-sided, and a *p* value less than 0.05 was considered statistically significant. Given the exploratory nature of the study and the multiple comparisons performed, the results are interpreted with emphasis on consistency and effect sizes rather than on isolated *p*-values (30, 31).

For logistic regression analyses, impaired HRQoL status was defined using a median split approach. The cut-off value corresponded to the median score of the study sample for each domain. Scores ≤ median were categorized as lower HRQoL, and scores > median as higher HRQoL.

### Ethical considerations

The institutional ethics committee in biomedical research at the University of Medicine and Pharmacy in Ho Chi Minh City approved the study protocol (IRB-VN01003/IRB00010293/FWA00023448; reference number 15/HĐĐĐ-ĐHYD). All participants were at least 18 years old, were informed about the study objectives and procedures, and provided written informed consent before data collection. Participation was voluntary, and patients were free to refuse participation, decline to answer individual questions, or withdraw from the study at any point without any impact on their clinical care.

## Results

### Patient inclusion and baseline characteristics

**Figure 1:**
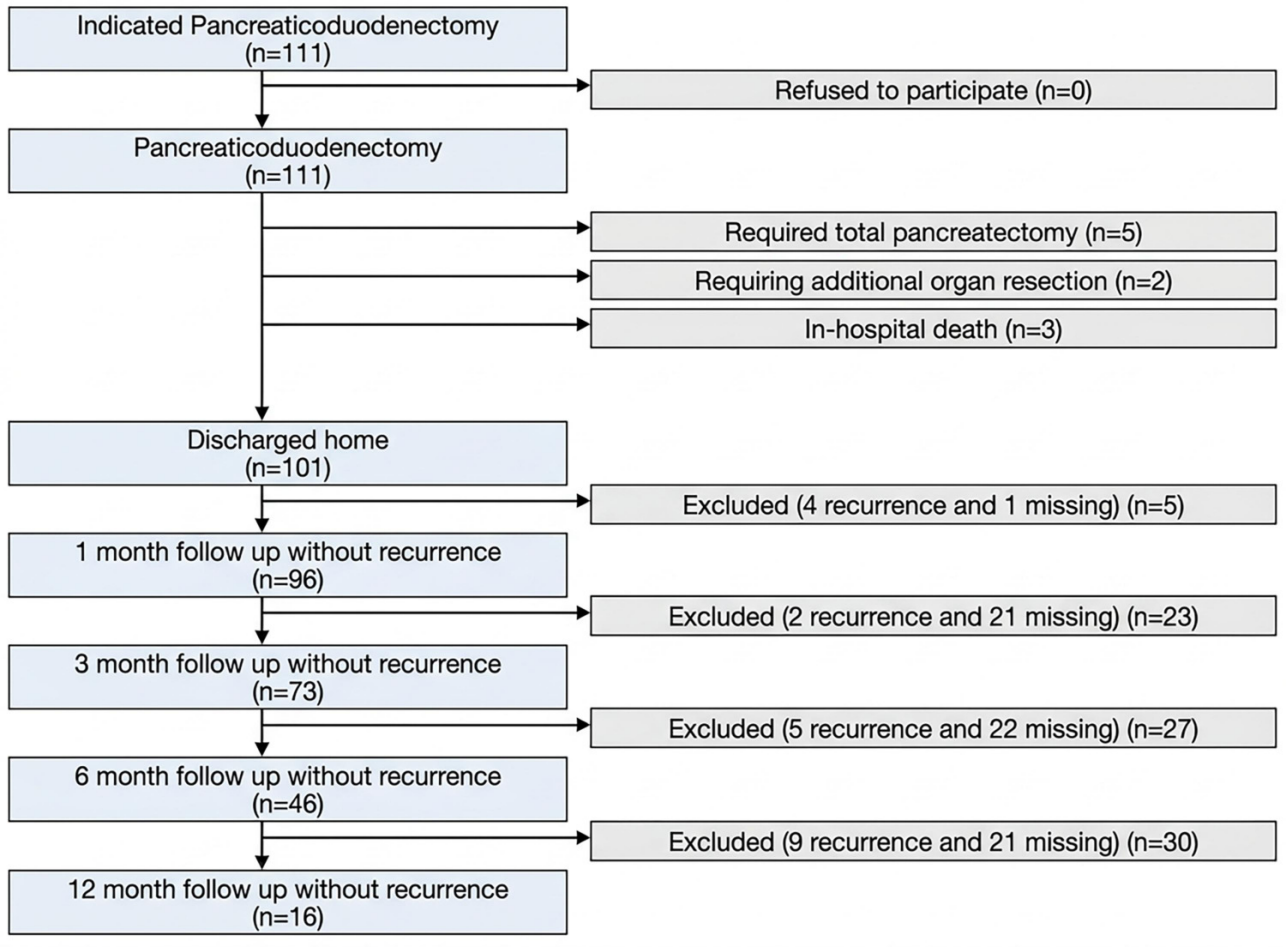
Flowchart describing patient cohort from inclusion to follow-up.

From January 2019 to May 2021, 111 patients that met the inclusion criteria underwent pylorus-preserving pancreaticoduodenectomy for periampullary tumors at the University Medical Center of Ho Chi Minh City. The patient flow, including exclusions and loss to follow-up at each time point, is summarized in Figure 1.

Among the 111 patients, 64 (57.7%) were male, and the mean age was 57.0 ± 12.2 years. Nutritional assessment showed that 23 patients (20.7%) were overweight, and 6 (5.4%) were moderately or severely underweight, although 21 patients (18.9%) required preoperative nutritional support due to acute weight loss. Jaundice was the predominant admission indication (58/111, 52.3%), followed by abdominal pain in 23 patients (20.7%). Cholangitis was present in 10 patients (9.0%). Biliary drainage was performed in 35 patients (31.5%) because of severe jaundice (total bilirubin >10 mg/dL) or cholangitis, mainly using endoscopic retrograde or percutaneous transhepatic biliary drainage (32, 33). Bilirubin levels before and afetr drainage are repored in Supplementary Table S3. A laparoscopic approach was used in 66 patients (59.5%), surrounding tissue invasion was identified in 23 patients (20.7%), most commonly involving the superior mesenteric vein (15/111, 13.5%) (34). Histopathology revealed pancreatic head adenocarcinoma in 44 cases (39.6%), ampullary cancer in 21 (18.9%), distal bile duct cancer in 14 (12.6%), and duodenal cancer in 8 (7.2%), with the remainder having benign or low-grade lesions. All patient characteristics are described in Table 1.

**Table 1:** Population characteristics.

Characteristic	*N* = 111
Gender	
Male	64 (57.7)
Female	47 (42.3)
Residence	
Urban	78 (70.3)
Rural	33 (29.7)
Occupation	
Manual labor	71 (64.0)
Intellectual labor	34 (30.6)
Unemployed	6 (5.4)
Comorbidities	
Hypertension	20 (18.0)
Diabetes	9 (8.1)
Hypertension and diabetes	10 (9.0)
COPD	2 (1.8)
Ischemic heart disease	1 (0.9)
Preoperative nutritional status	
Severe underweight (BMI <16)	2 (1.8)
Moderate underweight (16 ≤ BMI <17)	4 (3.6)
Mild underweight (17 ≤ BMI <18.5)	8 (7.2)
Normal weight (18.5 ≤ BMI <25)	74 (66.7)
Overweight (25 ≤ BMI <30)	22 (19.8)
Obesity (BMI >30)	1 (0.9)
Need for nutritional support	21 (18.9)
Reason of diagnosis	
Jaundice	58 (52.3)
Abdominal pain	23 (20.7)
Incidental	19 (17.1)
Vomiting	7 (6.3)
Melena	4 (3.6)
Presence of Cholangitis	10 (9.0)
Biliary drainage method	
No drainage	76 (68.5)
Endoscopic retrograde biliary drainage	16 (14.4)
Percutaneous transhepatic biliary drainage	16 (14.4)
Percutaneous transhepatic cholecystostomy	1 (0.9)
Biliary-enteric anastomosis	2 (1.8)
Time from biliary drainage to surgery (weeks)	
1	4 (3.6)
2	13 (11.7)
3	3 (2.7)
4	11 (9.9)
6	4 (3.6)
Postoperative histopathological diagnosis	
Pancreatic head cancer	44 (39.6)
Total pancreatic cancer	5 (4.5)
Other pancreatic tumors (IPMN, solid pseudopapillary tumor, neuroendocrine tumor, etc.)	16 (14.4)
Distal bile duct cancer	14 (12.6)
Ampullary cancer	21 (18.9)
Duodenal cancer	8 (7.2)
Hepatocellular carcinoma	1 (0.9)
Chronic pancreatitis	2 (1.8)
Surgical approach	
Laparoscopic	66 (59.5)
Open	45 (40.5)
Surrounding invasion	
None	88 (79.3)
Superior mesenteric vein	15 (13.5)
Pancreatic body	5 (4.5)
Liver	2 (1.8)
Hepatic artery	1 (0.9)
Pancreatic fistula	
None	77 (69.4)
Biochemical fistula	21 (18.9)
Grade B	12 (10.8)
Grade C	1 (0.9)

At baseline (V0), before surgery, mean SF-36 summary scores were 68.69 for the physical health component and 79.09 for the mental health component. Among physical domains, mean scores were 65.45 for physical functioning (PF), 63.60 for role-physical (RP), 82.88 for bodily pain (BP), and 62.84 for general health (GH), indicating moderate limitations in physical activities and general health despite relatively preserved pain control. Mental health domains showed higher baseline values, with mean scores of 66.08 for role-emotional (RE), 81.25 for social functioning (SF), 83.60 for vitality (VT), and 85.41 for mental health (MH), reflecting comparatively better preoperative emotional and social well-being. The scores are reported in full in Table 2.

**Table 2:** Average QoL scores before surgery (V0), and at 1 month (V1), 3 months (V3), 6 months (V6) and 12 months (V12) after discharge.

QoL domain	SF-36 total score (mean)				
	V0	V1	V3	V6	V12
Physical component summary (PCS)	68.69	68.14	80.98	87.07	101.97
Physical Functioning (PF)	65.45	66.05	71.03	76.91	84.69
Role Physical (RP)	63.60	54.69	79.11	89.67	87.50
Bodily Pain (BP)	82.88	85.20	94.68	97.61	97.25
General Health (GH)	62.84	66.61	79.11	84.46	82.19
Mental component summary (MCS)	79.09	77.76	89.85	93.41	92.22
Role Emotional (RE)	66.08	54.18	81.29	89.13	85.44
Social Functioning (SF)	81.25	81.16	91.84	94.39	95.19
Vitality (VT)	83.60	87.71	92.74	95.43	95.00
Mental Health (MH)	85.41	88.00	93.53	94.70	93.25

### Perioperative outcomes and survival

Postoperative pancreatic fistula occurred in 34 patients (30.6%), 21 (18.9%) had a biochemical fistula, 12 (10.8%) grade B fistula, and 1 (0.9%) with grade C fistula. The median length of hospital stay was 11 ± 5 days (range, 7–30 days), and in-hospital mortality was 2.7% (3 patients). Thirteen patients were readmitted before the first scheduled follow-up; the main reasons were intra-abdominal fluid collection and infection in 6 patients (46.2%) and enteritis in 4 (30.8%), while gastrointestinal bleeding, chronic anemia, and syncope each accounted for one readmission.

The 86 patients (77.5%) in which a malignant disease was found on histology were referred for oncological evaluation and were recommended treatment based on institutional protocols. 32 (28.8%) commenced chemotherapy within 1 month after discharge and additionally 14 patients started treatment within the following 9 months due to poor postoperative nutritional status, longer functional recovery, and the presence or severity of postoperative pancreatic fistula.

**Figure 2:**
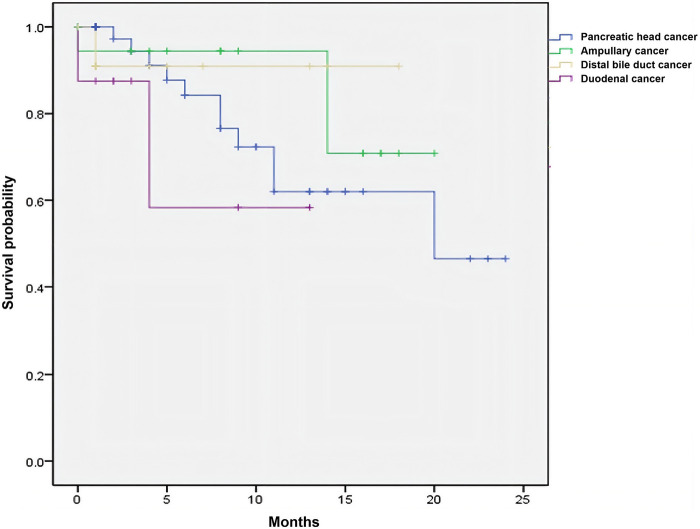
Kaplan–meier survival curves in months of patients with periampullary malignant tumors after pancreaticoduodenectomy, stratified by tumor type.

DFS at 1, 3, 6 and 12 month were 95.9%, 93.4%, 87.0% and 76.0% respectively, patients who experienced recurrence during follow-up were excluded from QoL analyses because of the expected strong impact of relapse on HRQoL, Figure 2.

### Longitudinal quality of life after pancreaticoduodenectomy

**Figure 3:**
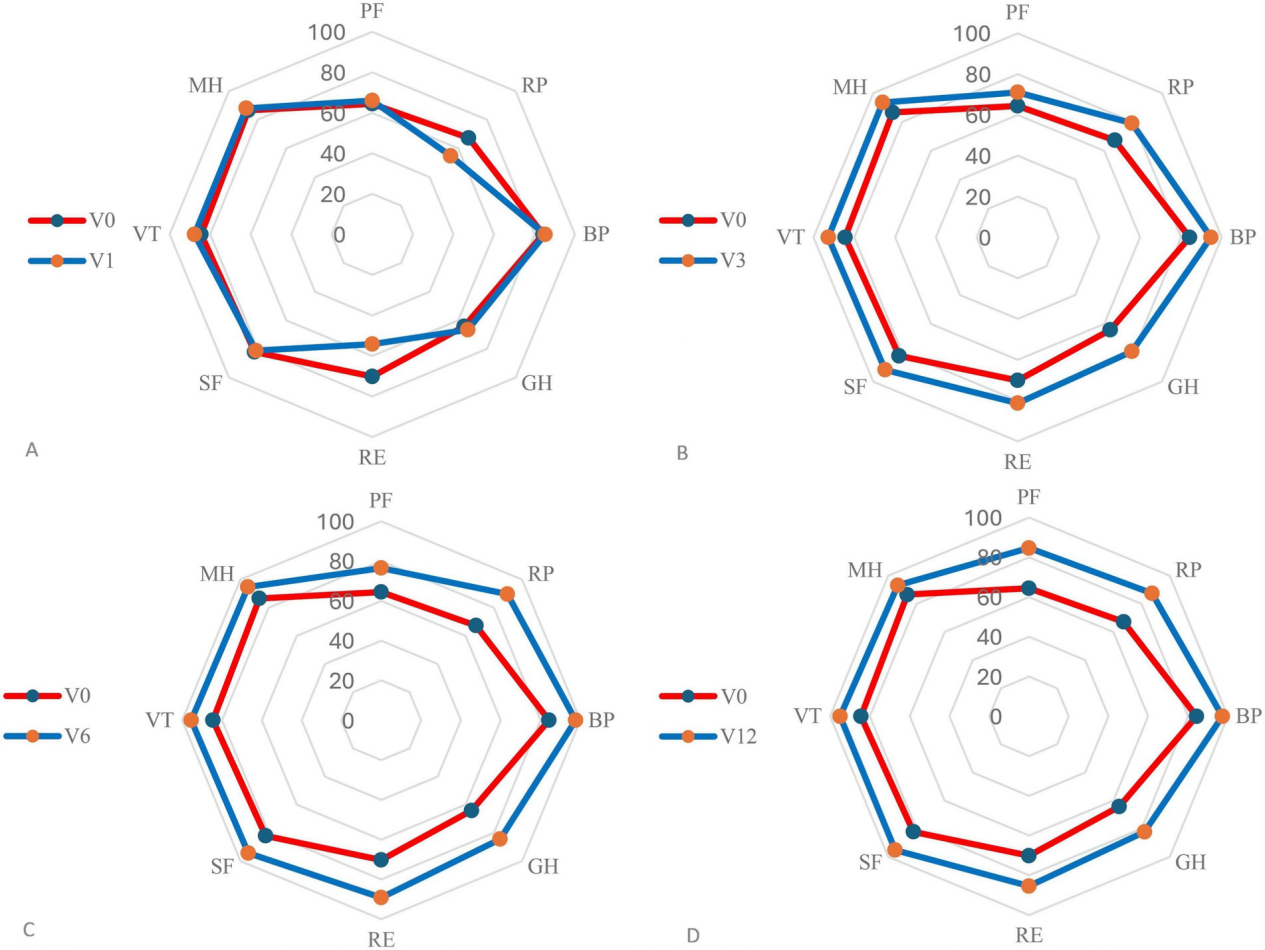
Comparison of QoL divided by domains (physical functioning (PF), role limitations due to physical health (RP), bodily pain (BP), general health perceptions (GH), vitality (VT), social functioning (SF), role limitations due to emotional problems (RE), mental health (MH))) at various time points: **(A)** before surgery (V0), compared to 1 month (V1), **(B)** before surgery (V0), compared to 3 months (V3), **(C)** before surgery (V0), compared to 6 months (V6) and **(D)** before surgery (V0), compared to 12 months (V12) after discharge. See Supplementary Table 4 for detailed scores comparision.

All 111 patients completed the SF-36 at baseline (V0). Follow-up completion declined over time, with cumulative loss to follow-up of 5 patients (4.95%) at 1 month (V1), 23 (23.96%) at 3 months (V3), 27 (36.99%) at 6 months (V6), and 30 (65.22%) at 12 months (V12) after discharge. Table 2 summarizes mean SF-36 scores at each time point, and Figure 3 illustrates their trajectories.

The physical health summary score remained stable at 1 month (68.69 at V0 vs. 68.14 at V1), but improved markedly thereafter to 80.98 at 3 months, 87.07 at 6 months, and 101.97 at 12 months, indicating sustained gains in overall physical functioning among those followed long-term. Within this component, role-physical decreased initially from 63.60 at baseline to 54.69 at 1 month, then rose to 79.11, 89.67, and 87.50 at 3, 6, and 12 months, respectively, while the other components increased steadily at each follow up. The mental health summary score showed a small, non-significant decline at 1 month (79.09 at V0 vs. 77.76 at V1), followed by improvement to 89.85 at 3 months, 93.41 at 6 months, and 92.22 at 12 months. Role-emotional mirrored the physical role pattern, decreasing from 66.08 at baseline to 54.18 at 1 month, then increasing to 81.29, 89.13, and 85.44 at 3, 6, and 12 months. Social functioning, vitality, and mental health increased from baseline, especially at later follow-ups. In formal tests, the early decreases in role-physical and role-emotional at 1 month were statistically significant compared with baseline (RP: *p* = 0.04; RE: *p* = 0.01). At the 12-month time point, only 16 patients remained in the study. Quality of life (QoL) was assessed in these 16 patients and compared with their own preoperative QoL. We acknowledge that the number of patients available at 12 months was very limited, with an attrition rate of 86.6%, which included **both** loss to follow-up and disease recurrence.

### Quality of life according to demographic and clinical characteristics

**Figure 4:**
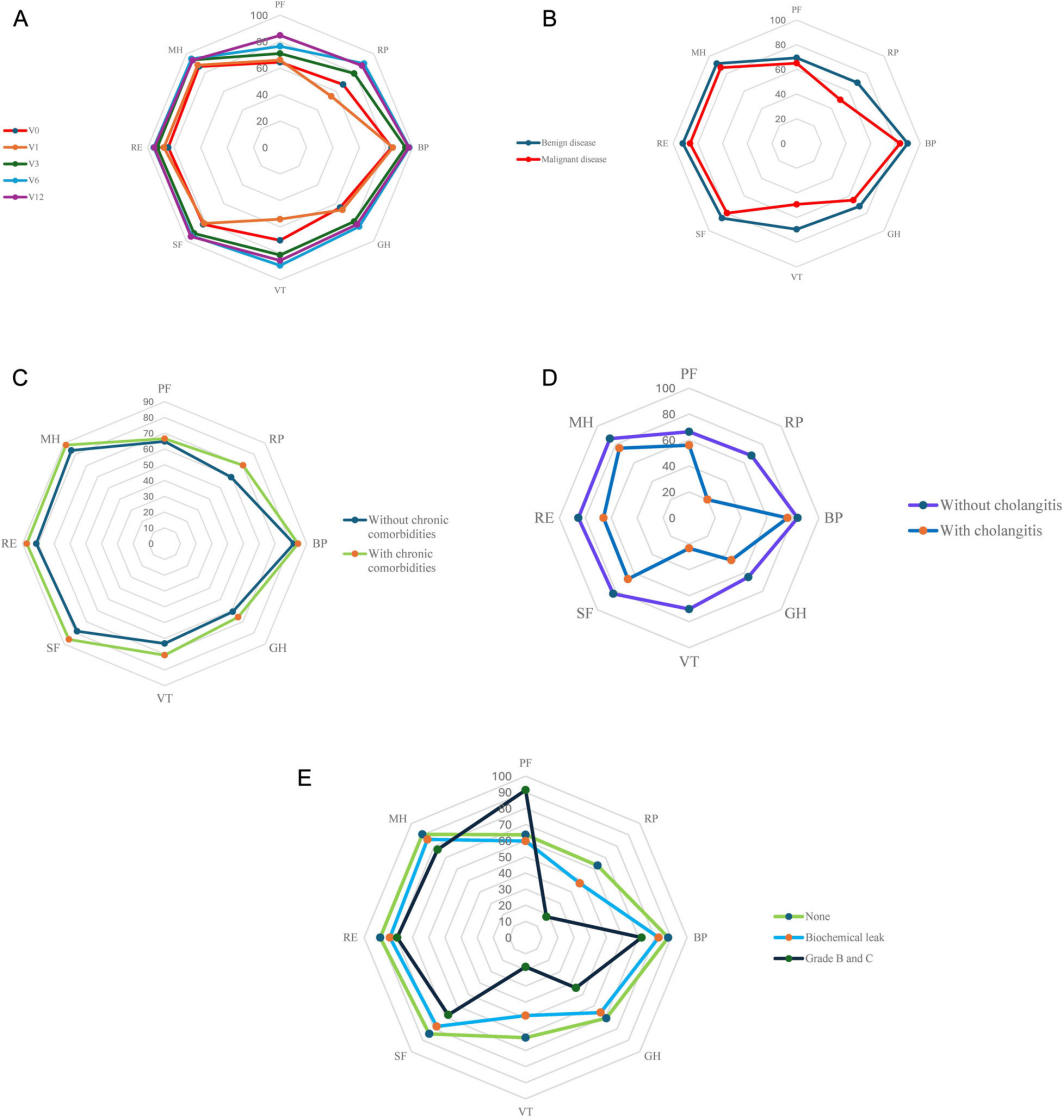
**(A)**. Comparison of QoL score, divided by domains (Physical Functioning (PF), Role Limitations due to Physical Health (RP), Bodily Pain (BP), General Health Perceptions (GH), Vitality (VT), Social Functioning (SF), Role Limitations due to Emotional Problems (RE), Mental Health (MH))) at various time points: before surgery (V0), and at 1 month (V1), 3 months (V3), 6 months (V6) and 12 months (V12) after discharge. **(B)**. Comparision of QoL score, divided by domains (Physical Functioning (PF), Role Limitations due to Physical Health (RP), Bodily Pain (BP), General Health Perceptions (GH), Vitality (VT), Social Functioning (SF), Role Limitations due to Emotional Problems (RE), Mental Health (MH))) between the benign and malignant disease histology groups. **(C)**. Comparision of QoL score, divided by domains (Physical Functioning (PF), Role Limitations due to Physical Health (RP), Bodily Pain (BP), General Health Perceptions (GH), Vitality (VT), Social Functioning (SF), Role Limitations due to Emotional Problems (RE), Mental Health (MH))) between patients with chronic comorbidities disease and those without. **(D)**. Comparision of QoL score, divided by domains (Physical Functioning (PF), Role Limitations due to Physical Health (RP), Bodily Pain (BP), General Health Perceptions (GH), Vitality (VT), Social Functioning (SF), Role Limitations due to Emotional Problems (RE), Mental Health (MH))) between patients with cholangitis at diagnosis and those without. **(E)**. Comparision of QoL score, divided by domains (Physical Functioning (PF), Role Limitations due to Physical Health (RP), Bodily Pain (BP), General Health Perceptions (GH), Vitality (VT), Social Functioning (SF), Role Limitations due to Emotional Problems (RE), Mental Health (MH))) between patients with pancreatic fistula divided by severity and those without (grade B and grade C fistula were reported in the same group). See Supplementary Table 5 for detailed scores comparision.

Before surgery, median PCS and MCS scores were significantly lower in older patients (>60 years old) compared with younger ones, with associated with reduced physical functioning, role-physical, role-emotional, vitality, and mental health domains. There were no significant differences in PCS or MCS between men and women, residence (urban vs. rural) and occupation (manual vs. intellectual labor) also did not show significant associations with preoperative PCS or MCS scores, despite possible underlying socioeconomic differences. Table 3 and Figure 4 present preoperative PCS and MCS scores according to demographic and clinical characteristics. Chronic comorbidities were strongly associated with impaired baseline QoL. Patients with at least one chronic condition had significantly lower PCS and MCS scores than those without comorbidities, with differences particularly evident in physical functioning, role-physical, bodily pain, vitality, and mental health domains, Figure 4C.

**Table 3:** Qol scores of physical component summary (PCS) and mental component summary (MCS) and their correlation with demographic characteristics of patients.

Factors	Physical component summary (PCS)		*p*	Mental component summary (MCS)		*p*
	Low	High		Low	High	
Gender			0.84			0.94
Male	23 (59%)	41 (56.9%)		14 (58.3%)	50 (57.5%)	
Female	16 (41%)	31 (43.1%)		10 (41.7%)	37 (42.5%)	
Age group			0.01			0.04
≤30	0 (0%)	5 (6.9%)		0 (0%)	5 (5.7%)	
31–40	1 (2.6%)	4 (5.6%)		1 (4.2%)	4 (4.6%)	
41–50	3 (7.7%)	13 (18.1%)		2 (8.3%)	14 (16.1%)	
51–60	11 (28.2%)	22 (30.6%)		6 (25%)	27 (31%)	
61–70	19 (48.7%)	24 (33.3%)		11 (45.8%)	32 (36.8%)	
>70	5 (12.8%)	4 (5.6%)		4 (16.7%)	5 (5.7%)	
Residence			0.54			0.35
Rural	13 (33.3%)	20 (27.8%)		9 (37.5%)	24 (27.6%)	
Urban	26 (66.7%)	52 (72.2%)		15 (62.5%)	63 (72.4%)	
Occupation			0.08			0.001
Unemployed	4 (10.3%)	2 (2.8%)		4 (16.7%)	2 (2.3%)	
Manual labor	26 (66.7%)	45 (62.5%)		17 (70.8%)	54 (62.1%)	
Intellectual labor	9 (23.1%)	25 (34.7%)		3 (12.5%)	31 (35.6%)	
Reason of diagnosis			0.02			0.01
Incidental	3 (7.7%)	16 (22.2%)		1 (4.2%)	18 (20.7%)	
Jaundice	21 (53.8%)	37 (51.4%)		13 (54.2%)	45 (51.7%)	
Abdominal pain	6 (15.4%)	17 (23.6%)		3 (12.5%)	20 (23%)	
Melena	4 (10.3%)	0 (0%)		3 (12.5%)	1 (1.1%)	
Vomiting	5 (12.8%)	2 (2.8%)		4 (16.7%)	3 (3.4%)	
Comorbidities			0.26			0.06
None	27 (69.2%)	42 (58.3%)		19 (79.2%)	50 (57.5%)	
Present	12 (30.8%)	30 (41.7%)		5 (20.8%)	37 (42.5%)	
BMI group			0.74			0.87
Severe underweight	1 (2.6%)	1 (1.4%)		0 (0%)	2 (2.3%)	
Moderate underweight	2 (5.1%)	2 (2.8%)		2 (8.3%)	2 (2.3%)	
Mild underweight	1 (2.6%)	7 (9.7%)		1 (4.2%)	7 (8%)	
Normal weight	26 (66.7%)	48 (66.7%)		15 (62.5%)	59 (67.8%)	
Overweight	8 (20.5%)	14 (19.4%)		6 (25%)	16 (18.4%)	
Obesity	1 (2.6%)	0 (0%)		0 (0%)	1 (1.1%)	
Need nutritional support			0.001			0.001
No	21 (53.8%)	69 (95.8%)		9 (37.5%)	81 (93.1%)	
Yes	18 (46.2%)	3 (4.2%)		15 (62.5%)	6 (6.9%)	
Preoperative cholangitis			0.03			0.15
No	32 (82.1%)	69 (95.8%		20 (83.3%)	81 (93.1%)	
Yes	7 (17.9%)	3 (4.2%)		4 (16.7%)	6 (6.9%)	
Pathology			0.001			0.99
Benign	2 (5.1%)	22 (30.6%)		0 (0%)	24 (27.6%)	
Malignant	37 (94.9%)	50 (69.4%)		24 (100%)	63 (72.4%)	

Nutritional status and the need for nutritional support were also correlated with QoL: patients requiring preoperative nutritional support had markedly lower PCS and MCS than those who did not (PCS: 46.2% vs. 4.2% in lower PCS category; MCS: 62.5% vs. 6.9%; both *p* < 0.001), reflecting the impact of malnutrition and frailty on perceived health. Preoperative cholangitis was associated with lower PCS scores (patients with cholangitis contributed 17.9% of low-PCS cases vs. 4.2% of high-PCS cases; *p* = 0.03), while its association with MCS did not reach statistical significance, Figure 4D.

Pathology had a particularly strong influence on PCS: in the low-PCS group, 94.9% of patients had malignant tumors, whereas only 69.4% of those with higher PCS had malignancy (*p* < 0.001). This difference was especially pronounced in role-physical and role-emotional, indicating a substantial physical burden of malignant disease even before surgery, Figure 4B. Postoperative complications, particularly pancreatic fistula, exerted a notable negative effect on early postoperative QoL. At 1 month after discharge, patients who developed pancreatic fistula had significantly lower physical functioning, role-physical, and role-emotional scores compared with those without fistula, with the steepest declines observed in patients with grade B leaks (Figure 4E). Patients without fistula or with only biochemical leaks showed a similar faster recovery in both physical and emotional domains.

### Multivariable analysis of factors associated with quality of life

In multivariable regression analyses, malignancy, chronic comorbidities, preoperative cholangitis, and postoperative pancreatic fistula remained independently associated with impaired QoL after adjustment for age, sex, and other clinical covariates, Table 4. Malignant pathology and the presence of comorbidities were the strongest predictors of lower PCS and MCS scores, and were particularly related to worse role-emotional and mental health domains, suggesting a combined physical and psychological burden in these patients. Preoperative cholangitis was independently associated with reduced physical functioning and general health, while postoperative pancreatic fistula, especially grade B, was mainly associated with lower physical functioning and role-physical scores in the early postoperative period.

**Table 4:** Multivariable analysis of factors affecting physical component summary (PCS) and mental component summary (MCS).

Physical Component Summary (PCS)	Univariable analysis			Multivariable analysis		
	OR	95% CI	*p*	OR	95% CI	*p*
Age group	0.566	0.376–0.853	0.007	0,578	0.340–0.984	0.044
Occupation	1.946	0.919–4.120	0.082	1.661	0.621–4.443	0.312
Reason of diagnosis	0.708	0.534–0.938	0.016	0.827	0.568–1.205	0.324
Preoperative nutritional support	0.051	0.014–0.189	0.0001	0.059	0.014–0.252	0.0001
Preoperative cholangitis	0.199	0.048–0.819	0.025	0.192	0.040–0.922	0.039
Benign/malignant disease	0.123	0.027–0.555	0.006	0.273	0.052–1.442	0.126
**Mental Component Summary (PCS)**	**Univariable analysis**			**Multivariable analysis**		
	**OR**	**95% CI**	***p***	**OR**	**95% CI**	***p***
Age group	0.615	0.384–0.985	0.043	0.649	0.338–1.245	0.193
Occupation	4.214	1.531–11.6	0.005	5.986	1.468–24.411	0.013
Reason for admission	0.68	0.5–0.925	0.014	0.752	0.474–1.192	0.225
Preoperative nutritional support	0.044	0.014–0.143	0.0001	0.049	0.012–0.208	0.0001
Preoperative cholangitis	0.370	0.095–1.438	0.151	0.286	0.051–1.602	0.154
Benign/malignant disease	0.123	0.027–0.555	0.006	0.000	0.052–1.442	0.998

## Discussion

Our findings would suggest that, for carefully selected patients with periampullary tumors, pancreaticoduodenectomy is compatible with meaningful recovery of health-related quality of life and, in several domains, improvement beyond preoperative levels. While short-term deterioration in role-based functioning was evident at 1 month, both physical and mental HRQoL improved substantially from 3 months onward, with particularly pronounced gains in pain, general health, social functioning, vitality, and mental health (35).

The observed early decline in role-physical and role-emotional domains is consistent with the physiological and psychological burden of major abdominal surgery and the immediate postoperative recovery period. Similar trajectories have been reported in large series, where physical and social functioning deteriorated during the first 1–3 months after pancreaticoduodenectomy but recovered by 3–6 months, with resolution of pain and fatigue over time. Notably, in the current cohort, bodily pain scores were relatively high even before surgery and improved further at all postoperative time points, suggesting effective relief of tumor-related pain and obstructive symptoms, which may partially offset the impact of surgical trauma. This is in line with earlier reports demonstrating near-normal long-term QoL and acceptable symptom burden after pancreaticoduodenectomy when compared with healthy controls or patients undergoing less extensive procedures (8, 36–38).

The progressive improvement of both physical and mental health summary scores from 3 to 12 months, with values exceeding baseline among patients remaining in follow-up, suggests that surgical resection, when combined with appropriate perioperative care, can yield substantial functional and psychological benefits over the medium term. Contemporary data from elderly and high-risk cohorts indicate that, despite higher morbidity and somewhat inferior survival in older patients, long-term QoL after pancreaticoduodenectomy is generally good, particularly among survivors without recurrence. The current findings reinforce this notion in a mixed-age, predominantly malignant periampullary population, and underscore the importance of viewing QoL recovery as a dynamic process rather than a static endpoint (38, 39).

Preoperative HRQoL was significantly worse in patients with malignant compared with benign or low-grade tumors across both physical and mental domains. This mirrors several prior studies showing that malignant pathology, particularly pancreatic ductal adenocarcinoma, is associated with lower baseline QoL and poorer long-term outcomes than benign disease or periampullary carcinomas. In the present cohort, malignancy remained an independent predictor of lower PCS and MCS in multivariable models, even after adjustment for age and comorbidities, highlighting the combined burden of tumor-related symptoms, systemic effects of cancer, and psychological distress. These findings support a paradigm in which QoL is considered a core oncologic outcome, particularly in advanced or high-risk malignancies where survival gains may be modest and treatment decisions must balance longevity with lived experience (36, 38–41).

Chronic comorbidities and the need for nutritional support were strongly associated with lower preoperative PCS and MCS, emphasizing the role of systemic health and frailty in shaping QoL trajectories. This is consistent with broader literature showing that older age, comorbidity burden, and impaired functional reserve are major determinants of postoperative QoL and long-term independence after pancreatic surgery. Preoperative cholangitis was linked to lower physical scores, reflecting the acute symptom burden and inflammatory stress associated with biliary sepsis. These observations collectively argue for comprehensive prehabilitation strategies, optimizing comorbidities, nutrition, and biliary drainage, to enhance both surgical safety and downstream HRQoL. Structured prehabilitation or enhanced recovery protocols could improve quality of life, further studies are needed to analyses whether these intervention could translate into measurable gains in SF-36 domains in this setting (40–43).

Our results suggest that postoperative complications, and pancreatic fistula in particular, were associated with worse early postoperative QoL, patients with grade B fistulas reported significantly lower physical functioning, role-physical, and role-emotional scores at 1 month, suggesting that prolonged length of stay the need for further procedures meaningfully interfere with both physical and psychological recovery. Recent similar series indicate that major complications can have a sustained impact on functional outcomes and patient-reported QoL, particularly when they prolong hospitalization, delay adjuvant therapy, or exacerbate malnutrition. In the present study, the detrimental effect of fistula appears most pronounced in the early months, with subsequent convergence of scores (13, 44, 45).

Taken together, the current results echo and extend prior prospective work demonstrating that patients who recover from pancreaticoduodenectomy generally achieve near-normal or improved QoL over time, even in predominantly malignant populations. Systematic reviews and recent narrative syntheses highlight a broadly similar pattern: short-term deterioration in physical and social functioning; gradual recovery by 3–6 months; and ultimately stable or improved QoL in survivors, with survival itself being the principal long-term driver of patient-reported outcomes.

The present findings, drawn from a single high-volume center with standardized pylorus-preserving techniques, support offering pancreaticoduodenectomy to appropriately selected patients, including those with significant symptom burden, when the procedure is technically feasible and oncologically justified. At the same time, the strong associations between malignancy, comorbidities, cholangitis, and impaired HRQoL underscore that surgical decision-making should integrate baseline QoL, frailty, and patient preferences rather than focusing exclusively on resectability and survival probabilities. Incorporating patient-reported outcomes into routine clinical follow-up and multidisciplinary decision-making could also facilitate more nuanced, individualized discussions about the risks and benefits of pancreaticoduodenectomy, especially in elderly or comorbid patients where the margin between survival gain and QoL compromise may be narrow (13, 38–40, 44).

### Strengths, limitations, and future directions

Key strengths of this study include its prospective design, standardized use of a validated generic QoL instrument at multiple postoperative time points, and detailed characterization of both tumor- and patient-related determinants of HRQoL. The focus on periampullary tumors and the uniform use of pylorus-preserving pancreaticoduodenectomy by a single surgeon limit heterogeneity in operative technique and case mix, facilitating clearer interpretation of QoL trajectories.

Several limitations must also be acknowledged. The high cumulative loss to follow-up, particularly at 12 months (81.1%), substantially reduces the precision and generalizability of late QoL estimates and introduces the possibility of survivor and attrition bias, as patients with worse clinical courses may have been less likely to complete follow-up questionnaires. The single-center design may restrict external validity to similar high-volume settings. In addition, no prior sample size calculation was performed, and the study was not powered to detect small but potentially meaningful differences in specific QoL domains or subgroups.

Future research should aim to validate these findings in larger, multicenter cohorts with more complete long-term follow-up, to integrate both generic and disease-specific QoL instruments. Interventional studies could also assess the effect of interventions such as prehabilitation, enhanced recovery pathways, and structured psychosocial support on HRQoL outcomes (43).

## Conclusion

The present study underscores the complex interplay of factors influencing QoL after pancreaticoduodenectomy. While patients experience an initial decline in physical and emotional well-being postoperatively, gradual recovery is observed across most domains within the first year. However, the presence of malignancy, postoperative complications, and comorbidities significantly modulates this trajectory, highlighting the need for personalized postoperative care strategies. Future research should focus on optimizing supportive interventions to enhance long-term QoL outcomes in this patient population.

## Data Availability

The original contributions presented in the study are included in the article/Supplementary Material, further inquiries can be directed to the corresponding author/s.
