# Supplementary material for: Factors associated with changes in quality of life after pancreaticoduodenectomy for periampullary tumors
Source: Front Surg. 2026 Apr 23;13:1797159. doi: 10.3389/fsurg.2026.1797159 (PMC13158528; doi:10.3389/fsurg.2026.1797159)
Supplement: Supplementary file 2 [file Table2.docx]

**Supplementary Table 2:** SF-36 Health status domains and scales and the interpretation of scores.

| Domain | No. of Items | Meaning of Score | |
| --- | --- | --- | --- |
|  |  | Low | High |
| **Physical component summary (PCS)** | |  |  |
| Physical Functioning (PF) | 10 | Severely limited in performing all physical activities, including basic self-care tasks such as bathing and dressing. | Able to perform all physical activities, including the most strenuous, without health-related limitations. |
| Role Physical (RP) | 4 | Experiences difficulty in work or other routine activities due to physical health problems. | Reports no difficulty in work or other routine activities due to physical health during the past four weeks. |
| Bodily Pain (BP) | 2 | Experiences very severe pain that is extremely limiting. | Reports no pain or functional limitation due to pain during the past four weeks. |
| General Health (GH) | 5 | Perceives personal health status as poor, with expectations of further decline. | Perceives personal health status as excellent. |
| **Mental component summary (MCS)** | |  |  |
| Role Emotional (RE) | 4 | Experiences difficulty in work or daily activities due to emotional problems. | Reports no interference with work or daily activities due to emotional problems during the past four weeks. |
| Social Functioning (SF) | 2 | Experiences frequent and marked interference with normal social activities due to physical or emotional problems. | Reports full participation in social activities without interference from physical or emotional problems during the past four weeks. |
| Vitality (VT) | 3 | Feels constantly fatigued and worn out. | Feels energetic and vigorous at all times during the past four weeks. |
| Mental Health (MH) | 5 | Feels persistently nervous or depressed. | Feels calm, happy, and peaceful at all times during the past four weeks. |
