# Supplementary material for: Factors associated with changes in quality of life after pancreaticoduodenectomy for periampullary tumors
Source: Front Surg. 2026 Apr 23;13:1797159. doi: 10.3389/fsurg.2026.1797159 (PMC13158528; doi:10.3389/fsurg.2026.1797159)
Supplement: Supplementary file 3 [file Table3.docx]

**Supplementary Table 3:** Bilirubin levels before biliary drainage and before surgery in the patient group that underwent biliary drainage preoperatively

| Bilirubin levels (mg/dl) | Median | Standard Deviation | Range |
| --- | --- | --- | --- |
| Before biliary drainage | 10.7 | 8.2 | 0.4 – 30.5 |
| Before surgery | 1.9 | 4.6 | 0.08 – 18.8 |
