# Supplementary material for: Factors associated with changes in quality of life after pancreaticoduodenectomy for periampullary tumors
Source: Front Surg. 2026 Apr 23;13:1797159. doi: 10.3389/fsurg.2026.1797159 (PMC13158528; doi:10.3389/fsurg.2026.1797159)
Supplement: Supplementary file 4 [file Table4.docx]

**Supplementary Table 3 (See Figure 3)**

| **Domain** | V0 | V1 | p-value | **95% CI** |
| --- | --- | --- | --- | --- |
| **Physical component summary (PCS)** | 70,52 | 68,14 | 0,47 | - 4,12 to 8,90 |
| Physical Functioning (PF) | 64,41 | 66,05 | 0,94 | -9,70 to 10,41 |
| Role Physical (RP) | 67,29 | 54,69 | 0,04 | 0,30 to 24,91 |
| Bodily Pain (BP) | 84,25 | 85,20 | 0,65 | - 5,13 to 3,23 |
| General Health (GH) | 64,17 | 66,61 | 0,52 | - 9,88 to 4,99 |
| ***Mental component summary (MCS)*** | 80,84 | 77,76 | 0,29 | - 2,61 to 8,76 |
| Role Emotional (RE) | 70,17 | 54,18 | 0,01 | 4,55 to 27,41 |
| Social Functioning (SF) | 82,13 | 81,16 | 0,69 | - 3,86 to 5,80 |
| Vitality (VT) | 84,48 | 87,71 | 0,18 | - 8,00 to 1,54 |
| Mental Health (MH) | 86,58 | 88,00 | 0,51 | - 5,62 to 2,78 |

| **Domain** | V0 | V3 | p-value | **95% CI** |  |
| --- | --- | --- | --- | --- | --- |
| ***Physical component summary (PCS)*** | 67,24 | 80,98 | 0,00 | - 21,50 to - 5,99 |  |
| Physical Functioning (PF) | 63,97 | 71,03 | 0,02 | - 12,80 to - 1,31 | |
| Role Physical (RP) | 62,47 | 79,11 | 0,03 | - 31,12 to - 2,17 | |
| Bodily Pain (BP) | 83,05 | 94,68 | 0,00 | - 17,09 to - 6,17 | |
| General Health (GH) | 59,45 | 79,11 | 0,00 | - 28,17 to - 11,45 | |
| ***Mental component summary (MCS)*** | 77,44 | 89,85 | 0,00 | - 19,40 to - 5,41 | |
| Role Emotional (RE) | 65,32 | 81,29 | 0,02 | - 29,82 to - 2,12 | |
| Social Functioning (SF) | 79,51 | 91,84 | 0,00 | - 18,59 to - 6,07 | |
| Vitality (VT) | 81,44 | 92,74 | 0,00 | - 16,95 to - 5,66 | |
| Mental Health (MH) | 83,51 | 93,53 | 0,00 | - 14,86 to - 5,19 | |

| **Domain** | V0 | V6 | p-value | **95% CI** |
| --- | --- | --- | --- | --- |
| ***Physical component summary (PCS)*** | 67,20 | 87,07 | 0,00 | - 29,25 to - 10,48 |
| Physical Functioning (PF) | 66,09 | 76,52 | 0,00 | -17,80 to - 3,08 |
| Role Physical (RP) | 60,00 | 89,67 | 0,00 | - 47,80 to - 12,08 |
| Bodily Pain (BP) | 82,50 | 97,61 | 0,00 | -21,00 to - 9,22 |
| General Health (GH) | 60,22 | 84,46 | 0,00 | - 34,35 to - 14,13 |
| ***Mental component summary (MCS)*** | 77,65 | 93,41 | 0,00 | - 23,99 to - 10,48 |
| Role Emotional (RE) | 65,24 | 89,13 | 0,00 | - 39,60 to - 8,18 |
| Social Functioning (SF) | 80,72 | 94,39 | 0,00 | - 21,62 to - 5,73 |
| Vitality (VT) | 81,52 | 95,43 | 0,00 | - 20,73 to - 7,10 |
| Mental Health (MH) | 83,13 | 94,70 | 0,00 | - 17,54 to - 5,60 |

| **Domain** | V0 | V12 | p-value | **95% CI** |
| --- | --- | --- | --- | --- |
| ***Physical component summary (PCS)*** | 81,13 | 101,97 | 0,17 | - 51,33 to 9,65 |
| Physical Functioning (PF) | 76,25 | 84,69 | 0,08 | -18,06 to - 1,87 |
| Role Physical (RP) | 84,38 | 87,50 | 0,79 | - 27,39 to 21,14 |
| Bodily Pain (BP) | 87,63 | 97,25 | 0,01 | -16,68 to - 2,57 |
| General Health (GH) | 76,25 | 82,19 | 0,45 | - 22,11 to 10,23 |
| ***Mental component summary (MCS)*** | 85,91 | 92,22 | 0,29 | - 18,60 to 5,98 |
| Role Emotional (RE) | 81,31 | 85,44 | 0,70 | - 26,51 to 18,27 |
| Social Functioning (SF) | 91,69 | 95,19 | 0,50 | - 14,25 to 7,25 |
| Vitality (VT) | 85,63 | 95,00 | 0,12 | - 20,73 to - 7,10 |
| Mental Health (MH) | 85,00 | 93,25 | 0,19 | - 21,48 to 2,73 |
