# Supplementary material for: Factors associated with changes in quality of life after pancreaticoduodenectomy for periampullary tumors
Source: Front Surg. 2026 Apr 23;13:1797159. doi: 10.3389/fsurg.2026.1797159 (PMC13158528; doi:10.3389/fsurg.2026.1797159)
Supplement: Supplementary file 5 [file Table5.docx]

**Supplementary Table 4 (See Figure 4)**

**Fig 4 a:** Comparison of QoL score, divided by domains (Physical Functioning (PF), Role Limitations due to Physical Health (RP), Bodily Pain (BP), General Health Perceptions (GH), Vitality (VT), Social Functioning (SF), Role Limitations due to Emotional Problems (RE), Mental Health (MH))) at various time points: before surgery (V0), and at 1 month (V1), 3 months (V3), 6 months (V6) and 12 months (V12) after discharge.

| **Domain** | V0 | V1 | p-value | **95% CI** |
| --- | --- | --- | --- | --- |
| **Physical component summary (PCS)** | 70,52 | 68,14 | 0,47 | - 4,12 to 8,90 |
| Physical Functioning (PF) | 64,41 | 66,05 | 0,94 | -9,70 to 10,41 |
| Role Physical (RP) | 67,29 | 54,69 | 0,04 | 0,30 to 24,91 |
| Bodily Pain (BP) | 84,25 | 85,20 | 0,65 | - 5,13 to 3,23 |
| General Health (GH) | 64,17 | 66,61 | 0,52 | - 9,88 to 4,99 |
| ***Mental component summary (MCS)*** | 80,84 | 77,76 | 0,29 | - 2,61 to 8,76 |
| Role Emotional (RE) | 70,17 | 54,18 | 0,01 | 4,55 to 27,41 |
| Social Functioning (SF) | 82,13 | 81,16 | 0,69 | - 3,86 to 5,80 |
| Vitality (VT) | 84,48 | 87,71 | 0,18 | - 8,00 to 1,54 |
| Mental Health (MH) | 86,58 | 88,00 | 0,51 | - 5,62 to 2,78 |

| **Domain** | V0 | V3 | p-value | **95% CI** |  |
| --- | --- | --- | --- | --- | --- |
| ***Physical component summary (PCS)*** | 67,24 | 80,98 | 0,00 | - 21,50 to - 5,99 |  |
| Physical Functioning (PF) | 63,97 | 71,03 | 0,02 | - 12,80 to - 1,31 | |
| Role Physical (RP) | 62,47 | 79,11 | 0,03 | - 31,12 to - 2,17 | |
| Bodily Pain (BP) | 83,05 | 94,68 | 0,00 | - 17,09 to - 6,17 | |
| General Health (GH) | 59,45 | 79,11 | 0,00 | - 28,17 to - 11,45 | |
| ***Mental component summary (MCS)*** | 77,44 | 89,85 | 0,00 | - 19,40 to - 5,41 | |
| Role Emotional (RE) | 65,32 | 81,29 | 0,02 | - 29,82 to - 2,12 | |
| Social Functioning (SF) | 79,51 | 91,84 | 0,00 | - 18,59 to - 6,07 | |
| Vitality (VT) | 81,44 | 92,74 | 0,00 | - 16,95 to - 5,66 | |
| Mental Health (MH) | 83,51 | 93,53 | 0,00 | - 14,86 to - 5,19 | |

| **Domain** | V0 | V6 | p-value | **95% CI** |
| --- | --- | --- | --- | --- |
| ***Physical component summary (PCS)*** | 67,20 | 87,07 | 0,00 | - 29,25 to - 10,48 |
| Physical Functioning (PF) | 66,09 | 76,52 | 0,00 | -17,80 to - 3,08 |
| Role Physical (RP) | 60,00 | 89,67 | 0,00 | - 47,80 to - 12,08 |
| Bodily Pain (BP) | 82,50 | 97,61 | 0,00 | -21,00 to - 9,22 |
| General Health (GH) | 60,22 | 84,46 | 0,00 | - 34,35 to - 14,13 |
| ***Mental component summary (MCS)*** | 77,65 | 93,41 | 0,00 | - 23,99 to - 10,48 |
| Role Emotional (RE) | 65,24 | 89,13 | 0,00 | - 39,60 to - 8,18 |
| Social Functioning (SF) | 80,72 | 94,39 | 0,00 | - 21,62 to - 5,73 |
| Vitality (VT) | 81,52 | 95,43 | 0,00 | - 20,73 to - 7,10 |
| Mental Health (MH) | 83,13 | 94,70 | 0,00 | - 17,54 to - 5,60 |

| **Domain** | V0 | V12 | p-value | **95% CI** |
| --- | --- | --- | --- | --- |
| ***Physical component summary (PCS)*** | 81,13 | 101,97 | 0,17 | - 51,33 to 9,65 |
| Physical Functioning (PF) | 76,25 | 84,69 | 0,08 | -18,06 to - 1,87 |
| Role Physical (RP) | 84,38 | 87,50 | 0,79 | - 27,39 to 21,14 |
| Bodily Pain (BP) | 87,63 | 97,25 | 0,01 | -16,68 to - 2,57 |
| General Health (GH) | 76,25 | 82,19 | 0,45 | - 22,11 to 10,23 |
| ***Mental component summary (MCS)*** | 85,91 | 92,22 | 0,29 | - 18,60 to 5,98 |
| Role Emotional (RE) | 81,31 | 85,44 | 0,70 | - 26,51 to 18,27 |
| Social Functioning (SF) | 91,69 | 95,19 | 0,50 | - 14,25 to 7,25 |
| Vitality (VT) | 85,63 | 95,00 | 0,12 | - 20,73 to - 7,10 |
| Mental Health (MH) | 85,00 | 93,25 | 0,19 | - 21,48 to 2,73 |

**Fig 4 b:** Comparision of QoL score, divided by domains (Physical Functioning (PF), Role Limitations due to Physical Health (RP), Bodily Pain (BP), General Health Perceptions (GH), Vitality (VT), Social Functioning (SF), Role Limitations due to Emotional Problems (RE), Mental Health (MH))) between the benign and malignant disease histology groups

| **Domain** | Benign | | Malignant | p-value | **95% CI** |
| --- | --- | --- | --- | --- | --- |
| ***Physical component summary (PCS)*** | | 84,71 | 63,47 | 0,00 | - 30,87 to -11,60 |
| Physical Functioning (PF) | | 76,76 | 61,63 | 0,00 | -22,75 to -7,52 |
| Role Physical (RP) | | 88,24 | 55,35 | 0,00 | - 52,57 to -13,21 |
| Bodily Pain (BP) | | 87,65 | 80,86 | 0,18 | - 16,80 to 3,22 |
| General Health (GH) | | 86,18 | 56,05 | 0,00 | - 39,52 to -20,74 |
| ***Mental component summary (MCS)*** | | 92,66 | 74,89 | 0,00 | - 26,21 to -9,34 |
| Role Emotional (RE) | | 88,24 | 59,71 | 0,00 | - 48,00 to -9,05 |
| Social Functioning (SF) | | 94,18 | 77,21 | 0,00 | - 23,54 to -10,40 |
| Vitality (VT) | | 95,29 | 79,88 | 0,00 | - 21,93 to -8,89 |
| Mental Health (MH) | | 92,94 | 82,74 | 0,00 | - 17,73 to -2,67 |

**Fig 4 c:** Comparision of QoL score, divided by domains (Physical Functioning (PF), Role Limitations due to Physical Health (RP), Bodily Pain (BP), General Health Perceptions (GH), Vitality (VT), Social Functioning (SF), Role Limitations due to Emotional Problems (RE), Mental Health (MH))) between patients with chronic comorbidities disease and those without

| **Domain** | With  Chronic comorbidities | Without Chronic comorbidities | p-value | **95% CI** |
| --- | --- | --- | --- | --- |
| ***Physical component summary (PCS)*** | 66,82 | 71,78 | 0,01 | - 15,22 to 5,29 |
| Physical Functioning (PF) | 64,78 | 66,55 | 0,03 | - 10,25 to 6,72 |
| Role Physical (RP) | 59,57 | 70,24 | 0,01 | - 29,23 to 7,88 |
| Bodily Pain (BP) | 81,90 | 84,50 | 0,58 | - 9,90 to 4,70 |
| General Health (GH) | 61,01 | 65,83 | 0,01 | - 16,66 to 7,02 |
| ***Mental component summary (MCS)*** | 76,67 | 83,06 | 0,02 | - 15, 83 to 3,05 |
| Role Emotional (RE) | 63,29 | 70,67 | 0,04 | - 25,27 to 10,52 |
| Social Functioning (SF) | 78,48 | 85,81 | 0,00 | - 15,26 to 0,59 |
| Vitality (VT) | 81,30 | 87,38 | 0,02 | - 14,33 to 2,18 |
| Mental Health (MH) | 83,59 | 88,38 | 0,07 | - 12,09 to 2,34 |

**Fig 4 d:** Comparision of QoL score, divided by domains (Physical Functioning (PF), Role Limitations due to Physical Health (RP), Bodily Pain (BP), General Health Perceptions (GH), Vitality (VT), Social Functioning (SF), Role Limitations due to Emotional Problems (RE), Mental Health (MH))) between patients with cholangitis at diagnosis and those without

| **Domain** | Without cholangitis | With cholangitis | p-value | **95% CI** |
| --- | --- | --- | --- | --- |
| ***Physical component summary (PCS)*** | 70,60 | 49,45 | 0,62 | 4,17 to 38,13 |
| Physical Functioning (PF) | 66,39 | 56,00 | 0.79 | - 3,85 to 24,63 |
| Role Physical (RP) | 67,92 | 20,00 | 0,02 | 17,65 to 78,20 |
| Bodily Pain (BP) | 83,58 | 75,80 | 0,21 | - 4,53 to 20,09 |
| General Health (GH) | 64,50 | 46,00 | 0,06 | - 1,30 to 38,31 |
| ***Mental component summary (MCS)*** | 81,17 | 58,03 | 0,00 | 7,63 to 38,66 |
| Role Emotional (RE) | 70,31 | 23,40 | 0,00 | 17,84 to 75,97 |
| Social Functioning (SF) | 82,69 | 66,70 | 0,02 | 2,71 to 29,28 |
| Vitality (VT) | 85,35 | 66,00 | 0,00 | 5,72 to 32,98 |
| Mental Health (MH) | 86,34 | 76,00 | 0,10 | - 1,98 to 25,78 |

**Fig 4 e:** Comparision of QoL score, divided by domains (Physical Functioning (PF), Role Limitations due to Physical Health (RP), Bodily Pain (BP), General Health Perceptions (GH), Vitality (VT), Social Functioning (SF), Role Limitations due to Emotional Problems (RE), Mental Health (MH))) between patients with pancreatic fistula divided by severity and those without (grade B and grade C fistula were reported in the same group)

| **Domain** | **Pancreatic fistula grading** | | | p-value _Levene_ | p-value _Anova_  p- value _Robust_ |
| --- | --- | --- | --- | --- | --- |
|  | None | Biochemical leak | Grade B and C |  |  |
| ***Physical component summary (PCS)*** |  |  |  |  |  |
| Physical Functioning (PF) | 63,69 | 59,80 | 91,36 | 0,01 | 0,61 |
| Role Physical (RP) | 63,08 | 47,50 | 18.18 | 0,11 | 0,01 |
| Bodily Pain (BP) | 88,32 | 82,40 | 71,82 | 0,1 | 0,01 |
| General Health (GH) | 70,69 | 65,75 | 44,09 | 0,50 | 0,01 |
| ***Mental component summary (MCS)*** |  |  |  |  |  |
| Role Emotional (RE) | 62,06 | 48,35 | 18,18 | 0,01 | 0,01 |
| Social Functioning (SF) | 84,40 | 77,95 | 67,82 | 0,56 | 0,01 |
| Vitality (VT) | 90,08 | 84,05 | 79,55 | 0.48 | 0,06 |
| Mental Health (MH) | 90,46 | 86,00 | 77,09 | 0,75 | 0,02 |
